# Neural correlates of text‐based emoticons: a preliminary fMRI study

**DOI:** 10.1002/brb3.500

**Published:** 2016-06-10

**Authors:** Ko Woon Kim, Sang Won Lee, Jeewook Choi, Tae Min Kim, Bumseok Jeong

**Affiliations:** ^1^ Clinical Neuroscience and Development LaboratoryGraduate School of Medical Science and EngineeringKorean Advanced Institute of Science and TechnologyDaejeonKorea; ^2^ Department of NeurologySamsung Medical CenterSungkyunkwan University School of MedicineSeoulKorea; ^3^ Department of PsychiatryDaejeon St. Mary's HospitalThe Catholic University of Korea College of MedicineDaejeonKorea; ^4^ Department of PsychiatryEulji UniversityDaejeonKorea

**Keywords:** Emoticon, emotion, fMRI, fusiform face area, occipital face area

## Abstract

**Introduction:**

Like nonverbal cues in oral interactions, text‐based emoticons, which are textual portrayals of a writer's facial expressions, are commonly used in electronic device–mediated communication. Little is known, however, about how text‐based emoticons are processed in the human brain. With this study, we investigated whether the text‐based emoticons are processed as face expressions using fMRI.

**Methods:**

During fMRI scan, subjects were asked to respond by pressing a button, indicating whether text‐based emoticons represented positive or negative emotions. Voxel‐wise analyses were performed to compare the responses and contrasted with emotional versus scrambled emoticons and among emoticons with different emotions. To explore processing strategies for text‐based emoticons, brain activity in the bilateral occipital and fusiform face areas were compared.

**Results:**

In the voxel‐wise analysis, both emotional and scrambled emoticons were processed mainly in the bilateral fusiform gyri, inferior division of lateral occipital cortex, inferior frontal gyri, dorsolateral prefrontal cortex (DLPFC), dorsal anterior cingulate cortex (dACC), and parietal cortex. In a percent signal change analysis, the right occipital and fusiform face areas showed significantly higher activation than left ones. In comparisons among emoticons, sad one showed significant BOLD signal decrease in the dACC, the left AIC, the bilateral thalamus, and the precuneus as compared with other conditions.

**Conclusion:**

The results of this study imply that people recognize text‐based emoticons as pictures representing face expressions. Even though text‐based emoticons contain emotional meaning, they are not associated with the amygdala while previous studies using emotional stimuli documented amygdala activation.

## Introduction

In the age of SNS, people communicate, share, and cooperate through social network services (SNS). The upsurge of text‐based communication (e.g., e‐mails, mobile text message, messages on the SNS) among people in modern society has drawn increasing attention to new forms of nonverbal communication. Text‐based emoticons are one of the most common forms of nonverbal communication in modern society, especially for young people. Accordingly, they have become as important as classic nonverbal communication such as facial expressions, gestures, and voice tone. Similar to facial expressions, a text‐based emoticon, as part of a message, functions as a nonverbal form of communication to deliver one's emotion, attitude, and intention (Derks et al. 2008a, 2007, 2008b,c). Therefore, it is important to comprehend processing strategies of text emoticons in the human brain. However, research on understanding the neurological mechanisms related to text‐based emoticons is strikingly lacking.

To date there have only been a handful of neuroimaging studies concerning text‐based emoticons. An fMRI study reported on the activation of the face‐specific area (right FFA) by graphic emoticons and faces but not by text‐based emoticons (Yuasa et al. 2011a). In contrast, an EEG study revealed that face‐specific component (N170) was activated by text‐based emoticons as well as faces (Churches et al. 2014). Although the aforementioned study did not perform source reconstruction for N170, the FFA is generally thought as the source of the face‐selective N170 response (Liu et al. 2002, 2010). Previous studies were not enough to determine how emoticons are processed in the main human cortical system for face processing. However, we intended to clarify if text‐based emoticons yield activation in face‐specific areas or not using fMRI.

The main human cortical system involved in face perception is composed of the fusiform face area (FFA), the occipital face area (OFA), and the superior temporal sulcus (STS) (Kanwisher et al. 1997; Gauthier et al. 2000; Haxby et al. 2000). Previous research with fMRI suggests that the OFA is involved in recognition of facial components (featural processing), whereas the FFA is involved in recognition of facial silhouettes (configural processing) (Pitcher et al. 2011). Meanwhile, the STS is responsible for processing changeable aspects of faces such as eye gaze and facial expression (Puce et al. 1998; Haxby et al. 2000; Winston et al. 2004; Nakajima et al. 2014). Previous researchers demonstrated that these face‐selective areas were larger and more frequently found in the right hemisphere (RH) than in the left hemisphere (LH) (Haxby et al. 1994; Puce et al. 1996; Gauthier et al. 2000; Rossion et al. 2003; Schiltz and Rossion 2006; Large et al. 2008; Pitcher et al. 2009, 2011; Nichols et al. 2010). Their findings of RH dominance in face recognition have been generally accepted in this field of study (Liu et al. 2010; Pitcher et al. 2011).

With regards to emotional processing, there are couple of studies that have documented that the right inferior frontal gyrus (IFG) is commonly activated by text‐based emoticons, graphic emoticons, and faces (Yuasa et al. 2011a,b,c). The IFG is known to be activated by the emotional valence decision task (Nakamura et al. 1999). In the case of facial expressions, the amygdala, the insula, and the ACC are known as the main brain regions for emotion processing (Phan et al. 2002). Furthermore, it was investigated that there were selective differences between activations depending on the type of emotions. For instance, happy, fearful, and sad faces specifically activated the amygdala, whereas angry or disgusted faces activated insula (Fusar‐Poli et al. 2009). But, there have been no studies to compare neural networks between various emotional emoticons.

Although there was both fMRI study using face or graphic emoticon and EEG study using text‐based emoticon, previous studies were not enough to make clear whether text‐based emoticon is processed by FFA. Also, no study has investigated the difference in brain responses to various emotions with text‐based emotion. The aims of this study were the following: (1) to determine whether face‐specific areas are activated by text‐based emoticons and (2) to compare between emotional emoticons and scrambled emoticons. We examined the following hypothesis by seeking the answer to this research question: We hypothesize that if face‐specific areas are evoked by text‐based emoticons, text‐based emoticons trigger face‐specific mechanisms. In contrast, unless face‐specific areas are activated, text‐based emoticons may be processed through separate system. Our study also evaluated the different patterns of brain activity according to the type of emotion being represented as well as the difference between emotional emoticons and scrambled emoticons.

## Methods

### Subjects

Eighteen subjects (age = 30.5 ± 6.6 years, M:F = 10:8) were recruited through the message board at Eulji University Hospital in Daejeon, Korea. Subjects were interviewed and screened to exclude any possible neurological and psychiatric diseases. They were all right handed. This study was approved by the Eulji University Hospital's Institutional Review Board. All subjects gave their written informed consent before participating in the study.

### Validation of text‐based emoticon stimuli

**Figure 1:**
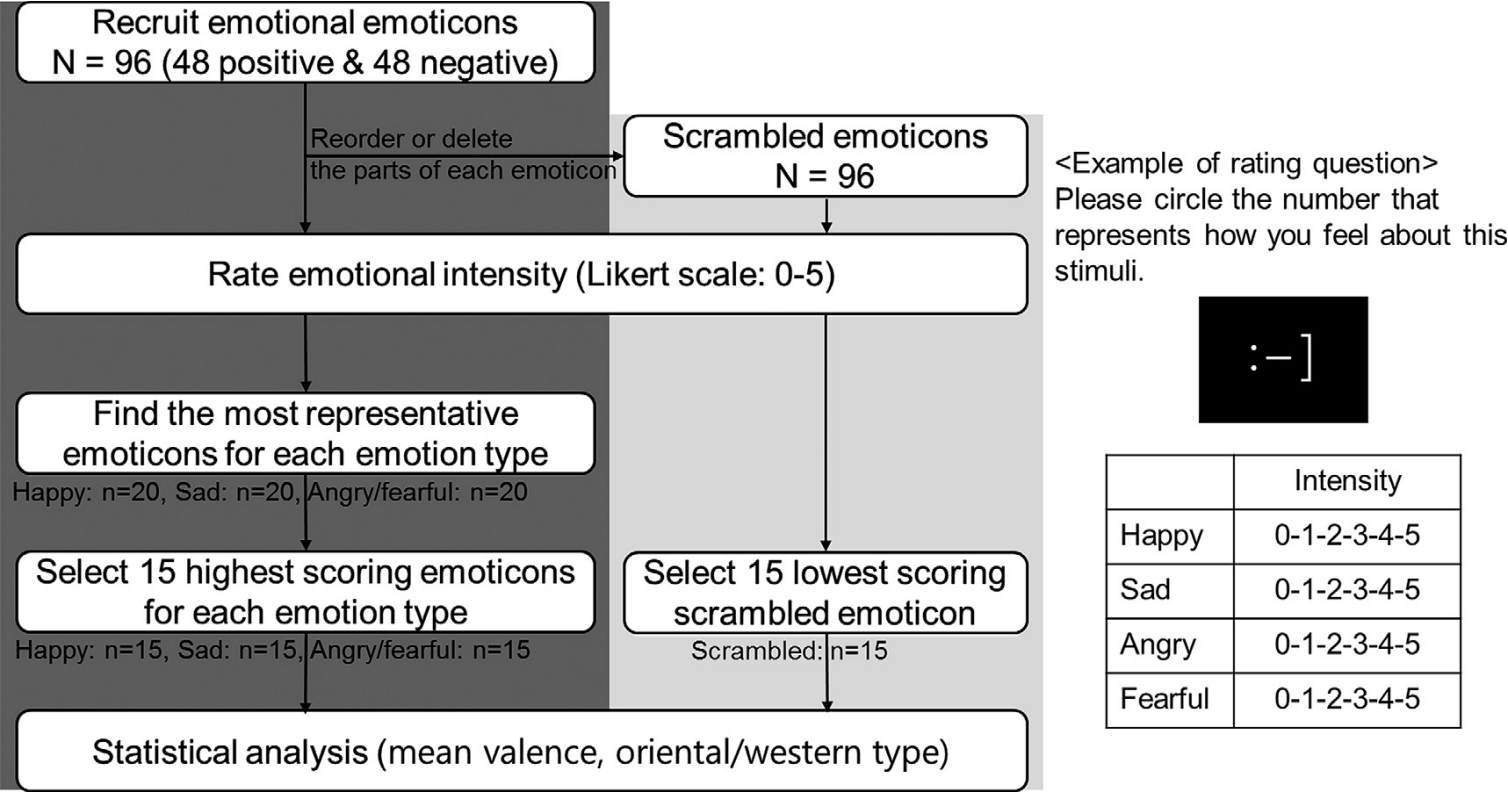
Standardization of text‐based emoticon stimuli. A semi‐automatic algorithm was used to find the most representative emoticons for each emotion type. A total of 48 positive and 48 negative emoticons were collected on the world‐wide web. Then we produced 96 scrambled emoticons which lose their configural information by reordering or deleting the parts of each positive or negative emoticon. The emotional valence of all 192 emoticons was measured on the Likert scale (0–5, 5 being the highest) for each condition by 20 healthy young volunteers (age = 20.8 ± 1.6 years, M:F = 13:7). Because only a limited number of emoticons satisfied our emotional valence criteria, both angry and fearful ones were assembled together into a single angry/fearful condition which consisted of five angry and 10 fearful emoticons. To control the emotional valence among emoticon conditions, the 15 highest scoring emoticons were selected for each emotional emoticon condition (happy, sad, and angry/fearful) and the lowest scoring 15 emoticons were chosen for scrambled emoticon condition.

We found the most representative text‐based emoticons for each emotion type and intensity through several steps (Fig. 1). The mean valence of the selected emoticons was not significantly different between the three emotional emoticons and was significantly lower for the scrambled emoticons than for the three emotional emoticons (*F*_(3, 77)_ = 111.44, *P* < 0.001). In this study, each of the emotional emoticons had both a vertical (oriental, erect) and horizontal (western, lean) orientation. Although subjects were familiar with both types, the linear‐by‐linear association test with frequency analysis of emoticon directions revealed that the percentage of the vertically and horizontally oriented emoticons significantly differed for each condition (*χ* ^*2*^ = 7.873, *P* = 0.049). Post hoc tests showed that the happy condition had significantly more horizontal‐oriented emoticons than angry/fearful ones (*χ* ^*2*^ = 6.533, *P* = 0.027). Accordingly, we used emoticon directions as a covariate while analyzing event‐related fMRI data.

### Experimental paradigm

**Figure 2:**
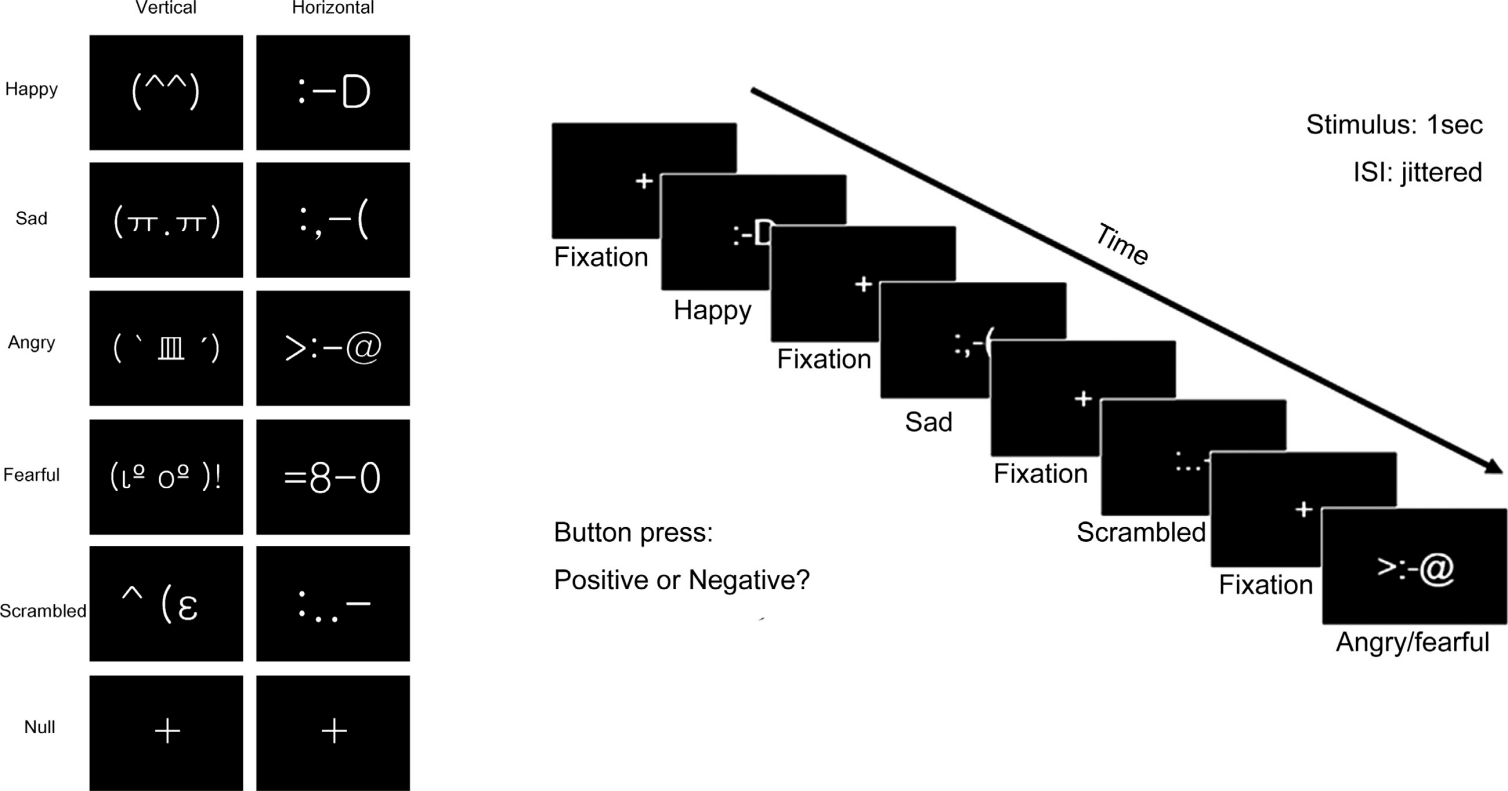
Text‐based emoticon stimuli and experimental paradigm. Both vertical (oriental, erect) and horizontal (western, lean) types of emoticons were used as stimuli. Each stimulus was presented for 1 sec with jittered interstimulus intervals (ISIs). Participants were asked to respond by pressing a button, indicating whether the emoticon represented a positive or negative emotion.

OptSeq2 RRID:SCR_014363** **(http://surfer.nmr.mgh.harvard.edu/optseq/), a tool for automatically scheduling events for rapid‐presentation event‐related fMRI experiments, was used for our event‐related paradigm (Fig. 2). With this software, the onset times of the events were jittered to remove the overlap from the estimate of the hemodynamic response. During a run of 310 sec, 40 null events as well as 15 emoticons for each four conditions (happy, sad, angry/fearful, and scrambled) were presented in pseudorandom order. Each stimulus was presented for 1 sec with jittered interstimulus intervals (ISIs) between 1 and 11 sec. During the fMRI scan, subjects were asked to respond by pressing a button indicating whether the emoticon represented a positive or negative emotion (Duncan et al. 2000). Scrambled emoticons were arbitrarily determined to be either a positive or negative emotion by each subject. For this reason, scrambled emoticons were subdivided into “scrambled positive” and “scrambled negative” conditions according to each answer.

### Statistical analyses for behavioral data

A Pearson's chi‐square test with frequency analysis was carried out to analyze the error rate among the happy, sad, and anger/fear emoticons. A one‐way ANOVA was performed to compare the mean response time among the four conditions and response errors; the error condition was defined as no responses, wrong responses, and delayed (>1.1 sec) responses.

### MRI data acquisition

All 18 subjects underwent MRI procedures on the 3.0‐T whole‐body MRI Echo speed system (ISOL, Korea). A high‐resolution structural MRI examination (TE = 5.7 msec, TR = 10 msec, field of view (FOV) = 220 mm, matrix size = 256 × 256, slice thickness = 1.5 mm, MPRAGE sagittal slices) was performed for each subject to exclude brain abnormalities. A total of 155 EPI scans of the blood oxygen level–dependent (BOLD) responses (TE = 35 msec, TR 2 sec, FOV = 192 × 220 mm, flip angle = 70°, slice thickness = 5.5 mm, no gap, 64 × 64 matrix, 25 axial slices) and also in‐plane T1‐weighted anatomical data (TE = 16 msec, TR = 2800 msec, FOV = 192 × 220 mm, matrix size = 192 × 256, flip angle = 60°, slice thickness = 5.5 mm, no gap, 25 axial slices) were collected for each participant. In addition to this emoticon experiment, two additional fMRI datasets were acquired from all subjects. Therefore, each of the subjects invested about 1 h to complete the entire MRI process.

### Processing and analysis of fMRI data

#### Acquisition and preprocessing

All images were processed and analyzed using the FMRI Expert Analysis Tool (FEAT) of FSL RRID:SCR_002823 (FMRIB's Software Library, version 5.0 http://fsl.fmrib.ox.ac.uk/fsl/fslwiki/FSL) (Smith et al. 2004; Woolrich et al. 2009; Jenkinson et al. 2012). The first four scans were discarded. The remaining 151 images were spatially realigned using rigid‐body transformation. Next, a brain mask from the first volume in the fMRI data was created for getting rid of signals outside each subject's brain. To reduce noise without reducing valid activation, spatial smoothing was performed using 5 mm FWHM, and a 50‐sec high‐pass filter was applied. Pre‐whitening, removal of serial correlations, was performed to make the statistics valid and maximally efficient. fMRI images were registered to the T1‐weighted structural image with translation (6 degrees of freedom), to the high‐resolution structural image with linear transformation (FLIRT) and standard space images using FSL nonlinear registration tool (FNIRT).

#### Statistical analyses for preprocessed fMRI data

After preprocessing, single and group‐level fMRI analyses were performed with FEAT in the FSL (Smith et al. 2004; Woolrich et al. 2009; Jenkinson et al. 2012). The general linear model (GLM) was used for linear combination of the modeled response to six visual stimulations, happy, sad, angry/fearful, scrambled positive, scrambled negative, and null (Fig. 2). The GLM was constructed of stimuli only showing the correct response for each subject. Errors were separated and gathered as another condition which was added as an independent variable into the above‐mentioned statistical model.

Head motion parameters were also included as confound variables in GLM. Activation maps for the contrasts, happy, sad, angry/fearful, scrambled positive, scrambled negative, and null, were constructed separately for each subject. A triple *t*‐test was performed to compare the activation among three emotional and two scrambled emoticon conditions. The type of orientation of the emoticons was added to the model as a covariate because both vertically and horizontally oriented emoticons were used in the experimental design. The resulting *Z* statistic image in each subject was entered to mixed‐effect analysis to show which clusters of voxels were activated at a significance level of *P* < 0.05 (FWE corrected). Activations were identified at a spatial extent of at least 10 voxels.

In order to compute percent signal change, regions of interest (ROIs) (the FFA and OFA) were defined as follows. First, we defined the group ROIs on functional grounds as a 5‐mm sphere centered on the mid‐fusiform gyrus and inferior occipital gyrus, which showed significantly higher activation to emotional emoticons compared to fixation cross. The STS was excluded because it was not localized by the contrast of emotional emoticons versus fixation. Second, we defined the individual ROIs using the group ROIs as a mask. Then the mean percent signal change data were extracted in the predefined ROIs: right OFA, left OFA, right FFA, and left FFA. Unpaired *t*‐tests were used to compare contralateral ROIs of each hemisphere.

## Results

### Behavioral tests

Behavioral data were acquired from only 14 of the proposed 18 subjects because of technical failure during the fMRI scan. Accuracy of task performance inside the scanner for emoticons was considered. A Pearson's chi‐square test showed that there was a trend‐level difference in error rates among three emotional emoticons (*χ* ^2^ = 5.718, *P* = 0.057). Post hoc analysis demonstrated that the trend‐level difference was caused by the lower error rate of sad, compared to happy, emoticons (*χ* ^2^ = 5.673, *P* = 0.017). In the fMRI analysis, trials showing error response were excluded from their corresponding conditions. A one‐way ANOVA showed significantly slower response times to the scrambled emoticon, compared to the emotional emoticon showing only error‐free responses (*F*_(4, 803)_ = 127.3, *P* < 0.001). The mean response time was not different among the three emotional conditions.

### Brain activity response to text‐based emoticons

#### Emotional versus scrambled emoticons

**Figure 3:**
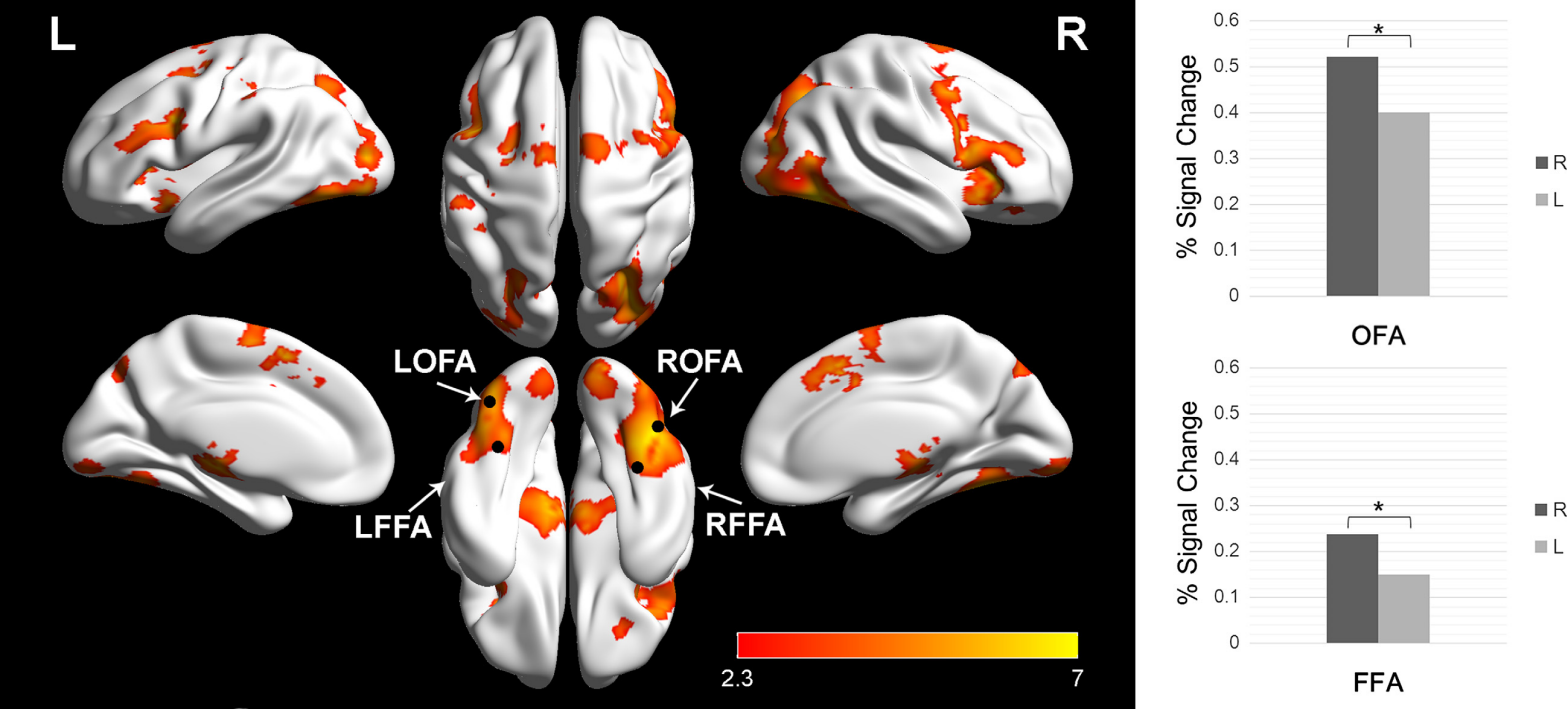
Emoticon processing network and comparison between right and left face areas. Yellow‐red clusters represent significant emoticon processing areas: three emotional conditions > null condition. These include the bilateral fusiform gyri, inferior division of lateral occipital cortex, inferior frontal gyri, dorsolateral prefrontal cortex (DLPFC), dorsal anterior cingulate cortex (dACC), and parietal cortex. Activations were also seen in bilateral thalamus, precuneus, and left precentral gyri. The mean percent signal change in the right OFA and FFA was significantly higher than the left OFA and FFA (Right OFA: 44, ‐64, ‐18; Left OFA: ‐38, ‐76, ‐14; Right FFA: 34, ‐44, ‐20; Left FFA: ‐34, ‐54, ‐20).

Both emotional and scrambled emoticons elicited the activation of similar brain regions, and there was no significant difference in their activity (Fig. 3). The most prominent clusters were in the bilateral fusiform gyri, inferior division of lateral occipital cortex, inferior frontal gyri, dorsolateral prefrontal cortex (DLPFC), dorsal anterior cingulate cortex (dACC), and parietal cortex. Activations were also seen in bilateral thalamus, precuneus, and left precentral gyri.

#### Comparisons among emoticons

**Figure 4:**
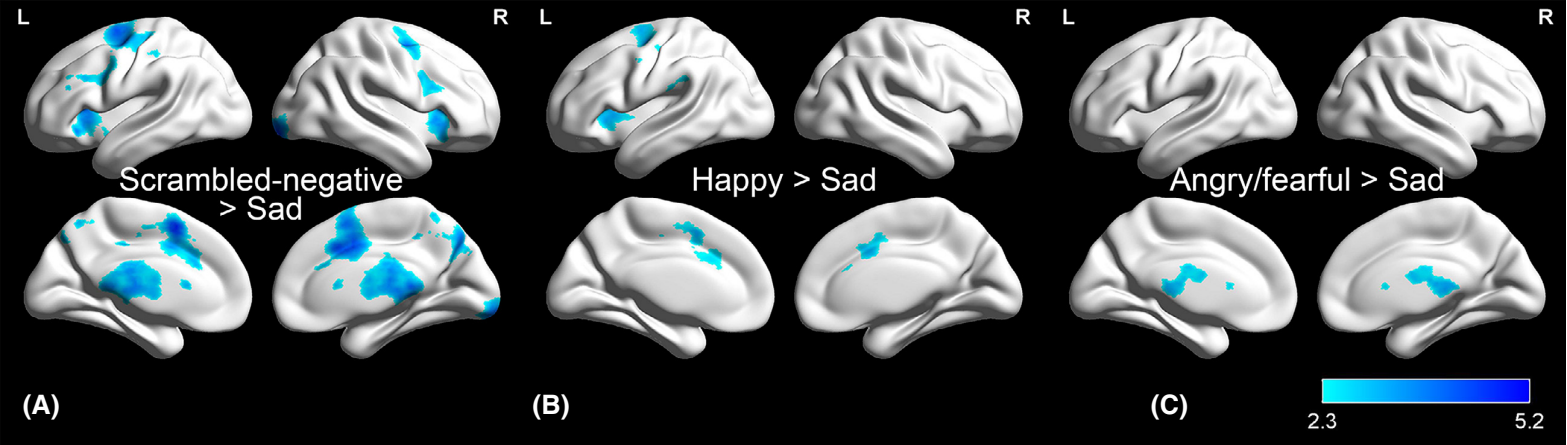
Differential emotional processing. Blue clusters show that there was decreased activation during processing of sad emoticons compared with other conditions. (A) The dorsal anterior cingulate (dACC), the anterior insula (AIC), the precuneus, and the occipital pole showed less activation in the sad than the scrambled negative condition. (B) The dACC, the left AIC, and the left precentral gyrus were less activated in the sad than happy condition; and (C) the bilateral thalamus demonstrated less activation in the sad than angry/fearful emoticons. No clusters were detected during the processing of other contrasts.

In spite of the similar activation maps between emotional and scrambled emoticons, several brain regions were less activated in the sad condition than other ones: happy, angry/fearful, and scrambled negative (Fig. 4). The comparison of happy versus sad yielded activation changes involving the dACC, the left AIC, and the left precentral gyrus (Fig. 4B). When the angry/fearful was contrasted with sad, there were activation changes in the bilateral thalamus (Fig. 4C). The comparison of scrambled negative versus sad elicited activation changes in the dACC, AIC, precuneus, and occipital pole (Fig. 4A). The result of scrambled positive versus sad was almost the same with that of scrambled negative versus sad.

#### Laterality of face‐specific areas

One of the questions in this study was whether the text‐based emoticons are processed mainly in the RH or LH. Emotional emoticons elicited the bilateral OFA and FFA activation. With the comparisons of mean percent signal changes between the RH and LH, the right FFA (M = 0.160, SD = 0.164) was higher than the left FFA (M = 0.075, SD = 0.149). This difference was significant, t _(82)_ = −2.499, *P* = 0.014. The mean percent signal change in the right OFA (M = 0.522, SD = 0.243) was significantly higher than the left OFA, t _(82)_ = 2.348, *P* = 0.021 (Fig. 3).

## Discussion

The object of this study was to investigate how the human brain recognizes text‐based emoticons. Our results demonstrated that text‐based emoticons are processed in the face‐specific areas: FFA and OFA. Other activations formed a bilateral network comprised of the following regions: inferior frontal gyri, DLPFC, dACC, and parietal cortex. Furthermore, our results showing significantly high percent signal changes in the right, compared with left, FFA and OFA suggest right hemisphere dominancy in information processing for emoticons.

In this study, we found that text‐based emoticons were processed in the face‐specific areas: FFA and OFA. This result implies that the text‐based emoticons are processed like facial expressions in the human brain. Not only emoticons but various other symbols, such as traffic signs and hazard warning symbols, have been used to convey ideas and beliefs. An fMRI study reported that icon processing was similar to that of pictures (Huang et al. 2015). Humans are widely capable of using symbolic representation in place of a concrete object and vice versa. That may be a uniquely human ability. The processing strategies of text‐based emoticons, symbolic representations of facial expressions, are thought to be similar to that of facial expressions.

Neuroimaging studies also have identified that the bilateral FFA and OFA are more activated to pictures of faces than objects with a RH dominance as our results (Kanwisher et al. 1997; McCarthy et al. 1997; Haxby et al. 1999; Rossion et al. 2000, 2003). Therefore, it is plausible that people recognize text‐based emoticons as pictures representing facial expressions. More recent neuroimaging studies support new hypotheses about the issue of laterality (Dien 2009). One of these new hypotheses is that the left FFA is associated with featural processing and the right FFA is associated with configural processing (Dien 2009).

Although our results from young adults suggested that usage of symbolic representation may be a human's unique ability, it remains unclear whether the ability is innate or not. This question might be addressed if our paradigm is applied to young children, ages of 5 or 6, who may not understand abstract concept (Cantlon et al. 2009). In the same vein, it is speculated that text‐based emoticons could elicit activation of FFA and OFA in elderly adults, although unfamiliarity may influence its intensity.

In our experiment, among core neural systems for face perception, the STS was not activated by emoticons. The activation of the STS has not been indicated in other emoticon studies (Shin et al. 2008; Yuasa et al. 2011a,c). Previous fMRI studies of face perception have shown that the STS is associated with the perception of facial components, the changeable aspect of a face, such as eye gaze and facial expression (Puce et al. 1998; Haxby et al. 2000; Winston et al. 2004). These changeable aspects of a face can be used to find clues about that person's state of mind (Hoffman and Haxby 2000). Furthermore, both fMRI and MEG studies examined the STS activation by turning heads compared to static faces (Lee et al. 2010). In addition, neurons responsive to facial expression were found in the STS of monkeys (Hasselmo et al. 1989). From that, we can infer that emoticons are too static and symbolic to activate the STS.

We ascertained that emotional and scrambled emoticons produced similar activation maps, although scrambled emoticons disrupted their configural processing. There are two important elements to explain these results. First, our scrambled emoticons contain featural information, whereas scrambled faces in previous studies (Jehna et al. 2011) do not contain discernible facial features. Scrambled emoticons were obtained by cutting out punctuation marks and by relocating these in non‐natural positions. Second, subjects were asked to respond by pressing a button indicating whether the emoticon represented a positive or negative emotion. Scrambled emoticons were arbitrarily determined to be either a positive or negative emotion because there was no button indicating neutral. For this reason, face‐specific areas might be activated while subjects tried to find emotional meaning. In the case of face studies, it was revealed that mental imagery of faces activated the FFA (O'Craven and Kanwisher 2000; Ishai et al. 2002).

Interestingly, there was no increased activation in the amygdala. This finding does not agree with other studies using emotional stimuli. It has been documented that the amygdala plays an important role in processing emotional stimuli from sources such as faces, scenes, words, and body expressions (Hadjikhani and de Gelder 2003; Maddock et al. 2003; Fusar‐Poli et al. 2009; Sabatinelli et al. 2011; Wallentin et al. 2011; Lindenberg et al. 2012). The amygdala with its connections to the affective division of the anterior cingulate cortex (ACC) is a central area for emotion perception (Devinsky et al. 1995; Whalen et al. 1998; Bush et al. 2000). In this study, the affective division of the ACC, which is associated with assessing emotional response (Phan et al. 2003), was not activated either. In contrast, recognition of emoticons elicited activations in the cognitive division of the ACC. This cognitive division of the ACC has a reciprocal interconnection with the DLPFC, which is responsible for executive functions (Bush et al. 2000). Our study implies that emotional processing of text‐based emoticons is less associated with the limbic system (Appendix S1). Further studies are, however, needed to explore the contribution of the emotional route to processing emoticons, as our experimental stimuli could not be salient enough to activate the limbic system.

Although emotional and scrambled emoticons evoked similar activation, sad emoticons elicited significantly decreased or underactivation in the dorsal anterior cingulate (dACC), the anterior insula (AIC), the precuneus, and the thalamus. This reduced activation during processing of sad emoticons can be explained as follows. Our behavior data show that sad had the lowest error rate, compared to other stimuli. Although trials showing error response were excluded from our fMRI analysis, sad responses would have been easier to recognize than other emotions. It is well known that the dorsal anterior cingulate (dACC) and the anterior insula (AIC) are involved in attention allocation to demanding stimuli (Menon and Uddin 2010; White et al. 2014). Consequently, it is reasonable to infer that the sad condition was more easily recognized, and so required less utilization of brain resources than other stimuli. Similarly, but not identically, sad faces have elicited limited activation in the amygdala relative to other emotional faces (Phillips et al. 1998; Kesler et al. 2001; Killgore and Yurgelun‐Todd 2004; Fusar‐Poli et al. 2009). In the case of both faces and emoticons, it is consistent that the sad condition evokes less brain activation than other emotions. In addition, this observation is consistent with the previous study mentioned above, where icons were associated more with the neocortex, whereas real faces are more related to the limbic system (Shin et al. 2008). On the other hand, activation of the thalamus angry/fearful versus sad condition may be related to differences in perceptual load, using selective attention (Kastner et al. 2004; Saalmann and Kastner 2011).

As mentioned in the above section, sad emoticons had the lower error rate than happy emoticons. It is unusual that happy faces have more errors in rating than negative faces. In our perspective, the reason why happy emoticons have more errors than negative emoticons is that happy condition had significantly more horizontal (western, lean) emoticons than negative ones. Although subjects could interpret both vertical (oriental, erect) and horizontal (western, lean) type of emoticons, they might be more familiar with vertical (oriental, erect) emoticons because subjects were all Asian. Accordingly, we used emoticon directions as a covariate while analyzing event‐related fMRI data.

There are some limitations to this study. Each of the emotional emoticons had both a vertical (oriental, erect) and a horizontal (western, lean) orientation. Subjects were familiar with both types and we used emoticon types as a covariate while analyzing event‐related fMRI data to avoid the disturbance factor. Nonetheless, this design may exert a complex influence on experimental results. Another limitation is that the fMRI paradigm did not have neutral emoticons. It should have additionally included neutral emoticons, as well as a neutral option for the behavioral rating of the emoticons. A third limitation is the subject's personal experience with using emoticons was not evaluated. The other limitation is that emoticons and faces were not directly compared in our study. To determine whether emoticons are interpreted as facial expressions, the best possible contrast would have been against real faces. However, the contrasts in this study were emotional emoticons versus scrambled emoticons. In addition, fixation cross‐mark may be less appropriate as a baseline for analysis at later stages of the visual streams which tap high‐level cognitive functions such as emoticon perception (Penny et al. 2007; Liu 2012). An emoticon versus face one‐to‐one comparison could help to explain emotional information processing in the human brain.

## Conclusion

In spite of these limitations, our study revealed that the face‐specific mechanism is involved in text‐based emoticon processing. Additionally, emoticons are processed mainly in cortical rather than limbic regions. Sad emoticons elicited significantly decreased activation or underactivation. Recognizing the meaning of stimuli is a fundamental cognitive process of the human brain. However, the question of how the human brain conceives meaning is not fully understood. Human brains have the unique ability to create contextual meanings during interactions with information perceived in a given situation. Further experiments could help advance research on this extraordinary human ability.

## Conflict of Interest

The authors have no conflicts of interest or financial interests, avoiding identifying any of the authors prior to peer review.

## Supporting information


**Appendix S1.** Supplemental results.
