# Supplementary material for: Neural correlates of text‐based emoticons: a preliminary fMRI study
Source: Brain Behav. 2016 Jun 10;6(8):e00500. doi: 10.1002/brb3.500 (PMC4980471; doi:10.1002/brb3.500)
Supplement: Supplementary file 1 — Appendix S1. Supplemental results. [file BRB3-6-e00500-s001.docx]

**Supplemental results**

In order to compute percent signal change, regions of interest (ROIs) were defined as follows. First, we defined the ROIs on functional grounds as a 5 mm-sphere centered which showed significantly higher activation to emotional stimuli in the meta-analytic coactivation analysis ([Yarkoni, et al., 2011](#_ENREF_1)). We used several key words to perform term-based analyses using Neurosynth: face, facial expression, emotional, and emotion regulation. NeuroSynth replicated previous findings of category-specific activation in regions such as the bilateral insula, dACC, amygdala, IFG, DLPFC, SPL, and STS. Second, we defined the individual ROIs using the group ROIs as a mask. Then the mean percent signal change data were extracted in the predefined ROIs. Repeated measure ANOVA were used to compare between emotional and scrambled emoticons.

**Abbreviations**: dACC, dorsal anterior cingulate cortex; IFG, inferior frontal gyrus; DLPFC, dorsolateral prefrontal cortex; SPL, superior parietal lobule; STS, superior temporal sulcus


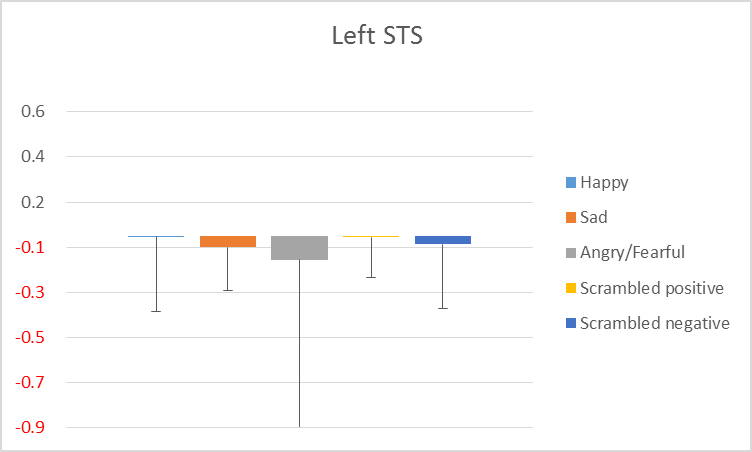


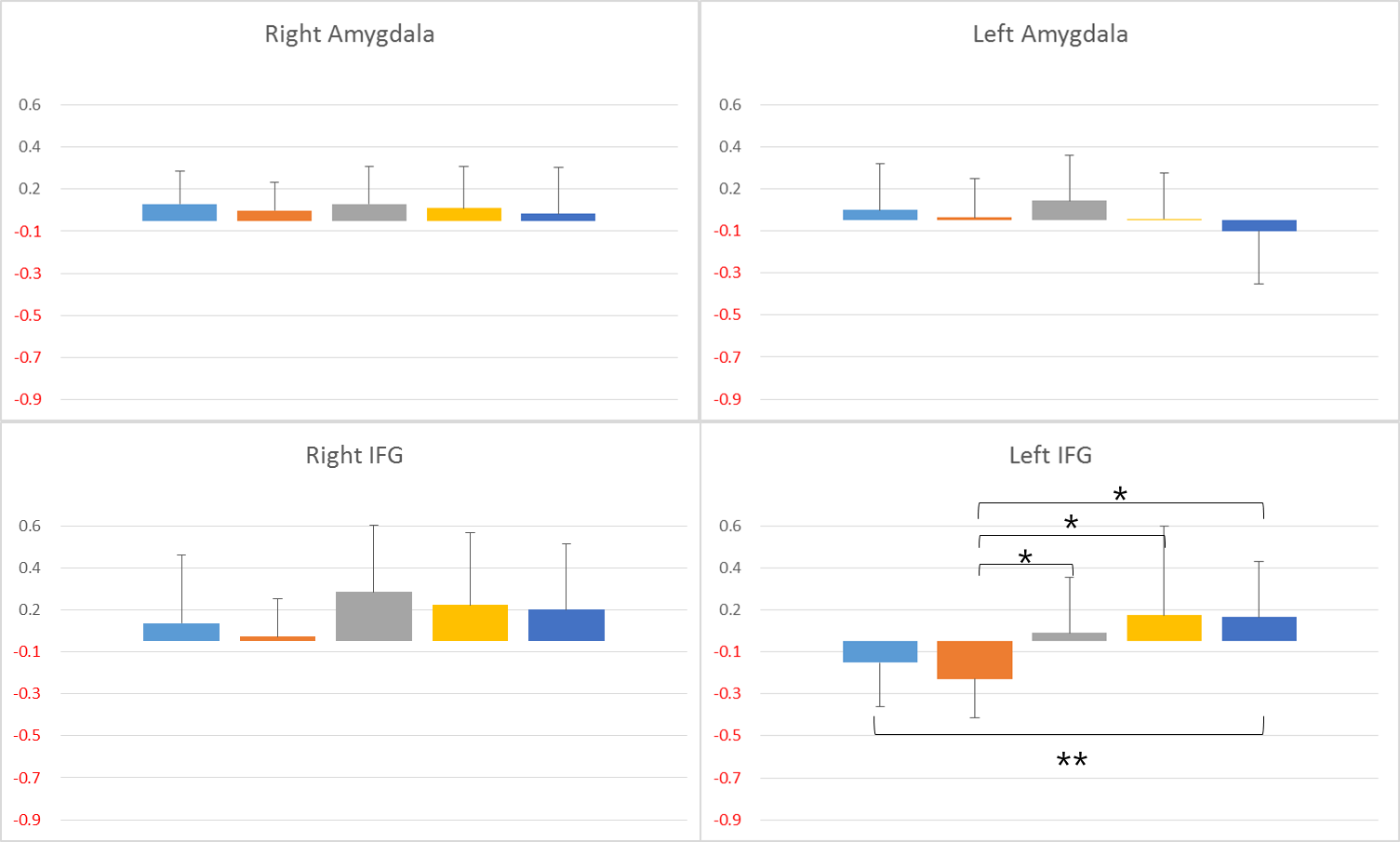


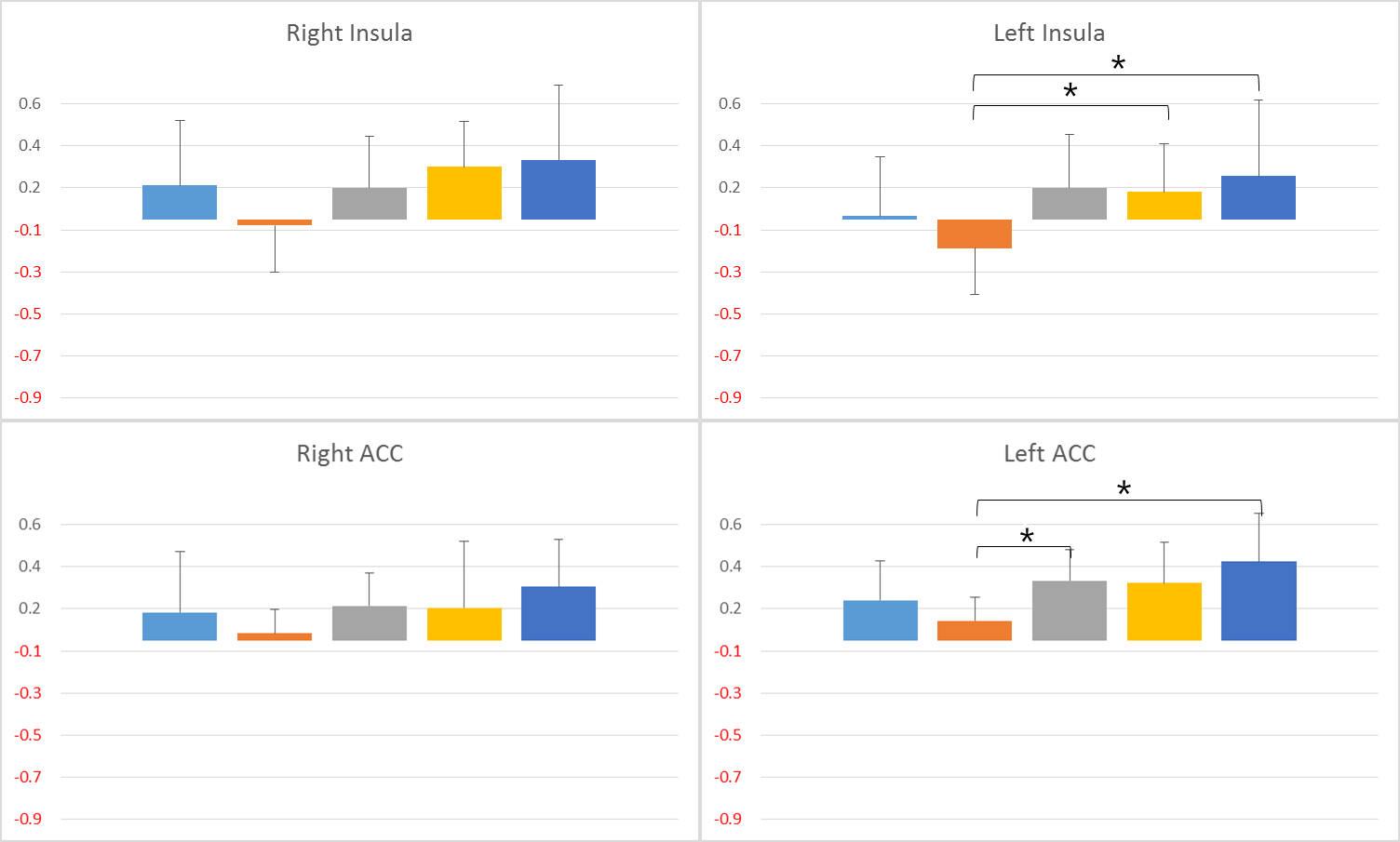


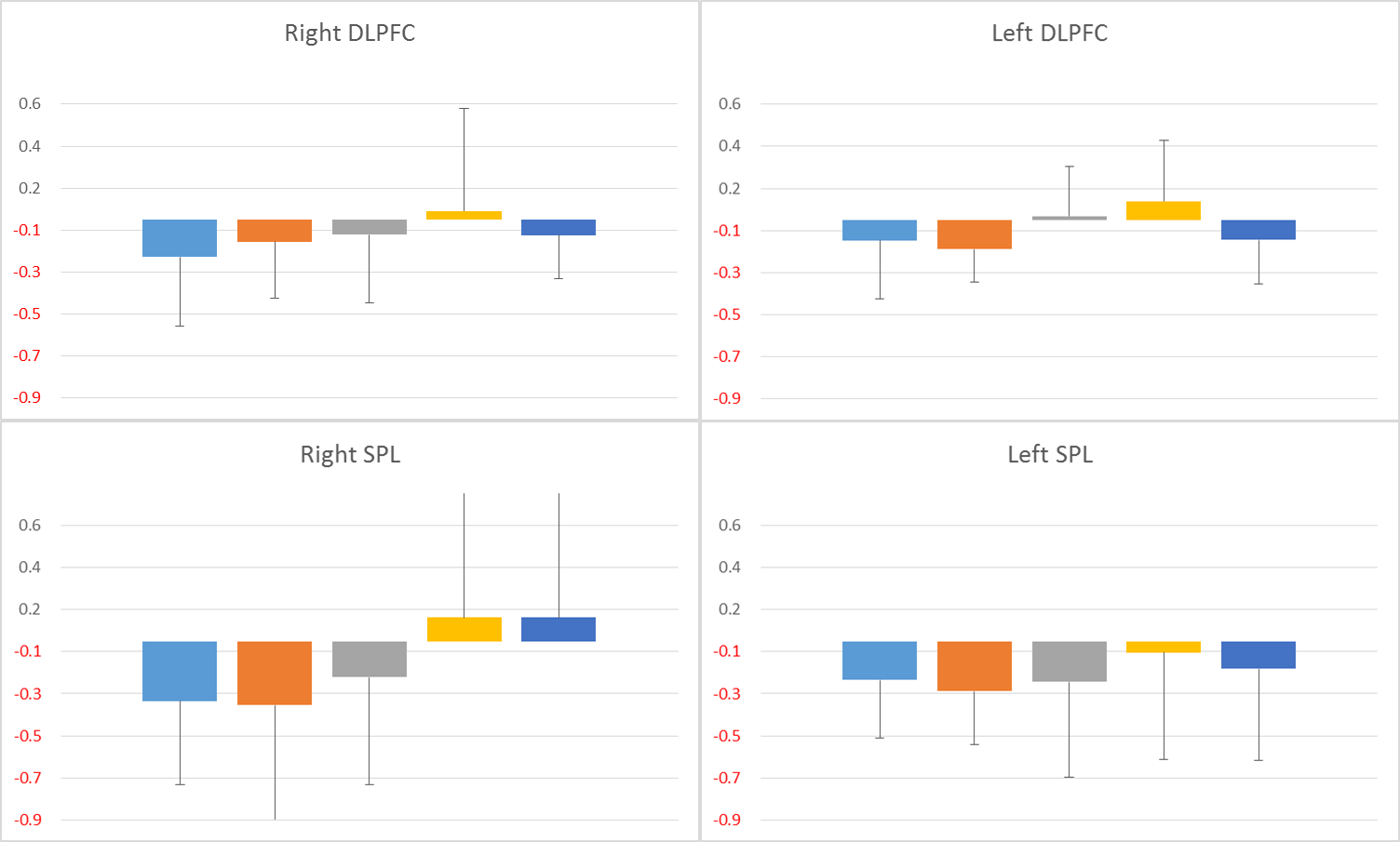


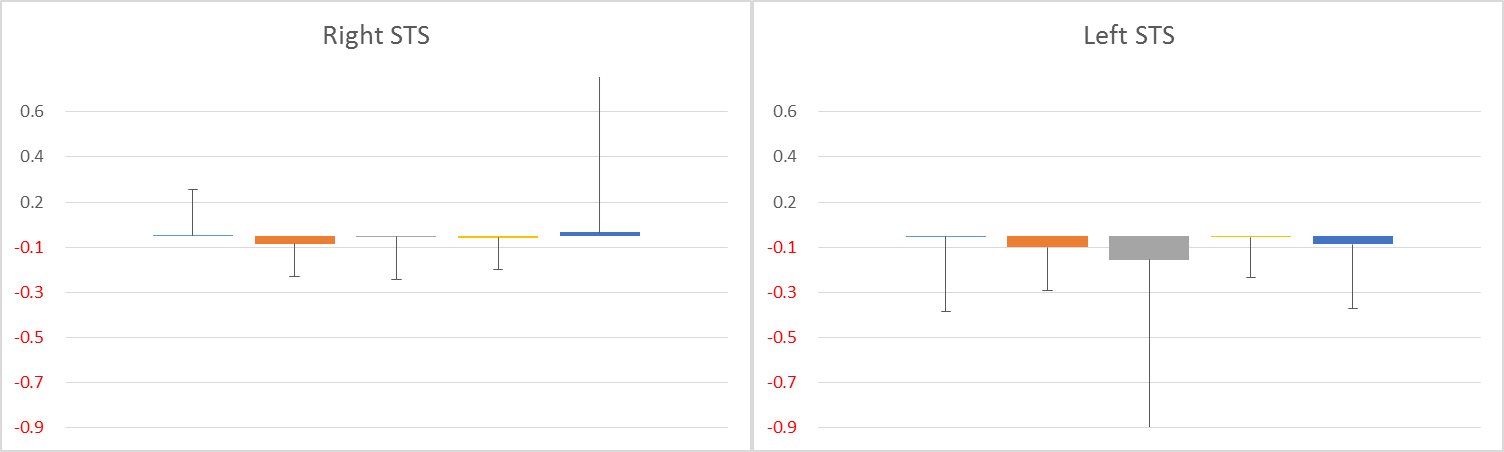


Yarkoni, T., Poldrack, R.A., Nichols, T.E., Van Essen, D.C., Wager, T.D. (2011) Large-scale automated synthesis of human functional neuroimaging data. Nature methods, 8:665-70.
